# Essay on Cataract

**Published:** 1859-02

**Authors:** 


### ESSAY ON CATARACT.

We are in recipt of "An Essay" on the treatment of Cataract, by Mark Stephenson, M. D., Surgeon to the New York Opthalmic Hospital, &c., &c., read before the American Medical Association at Washinington City, May 6th, 1858.

In this little pamphlet, we have discussed the various operations proposed for the relief of this important affection.

Differing from many Opthalmic Surgeons whose position in the scientific world are much less enviable, he has no favorite method to defend, but takes a rational and unbiased view of the various modes, contending that "the selection of the method best adapted to each case, constitutes in the majority of instances, the secret of success."

Had we fewer enthusiasts in our noble science, who strenuously contend for favorite methods to the exclusion of all others, much more would be accomplished for science and suffering humanity.

Dr. S. discusses at some length the contested point, as to whether any operation should be performed when an eye is sound, and is decidedly of opinion that in the majority of such cases an operation is demanded. The objection often urged, that the removal of the lens under such circumstances will result in double vision, he contends is fallacious, giving his own experience as well as that of other eminent Surgeons both in Europe and America, in opposition to such a result.

All who feel an interest in the treatment of cataract, would do well to procure a copy of this Essay, as it contains a review of the whole subject without, as before suggested, any partially for a favorite method.

The Essay may be had of the Apothecary of the New York Opthalmic Hospital, No. 6, Stuyversant street. Price 25 cents.

